# Neuroanatomical Characterisation of the Expression of the Lipodystrophy and Motor-Neuropathy Gene *Bscl2* in Adult Mouse Brain

**DOI:** 10.1371/journal.pone.0045790

**Published:** 2012-09-25

**Authors:** Alastair S. Garfield, Wai S. Chan, Rowena J. Dennis, Daisuke Ito, Lora K. Heisler, Justin J. Rochford

**Affiliations:** 1 Department of Pharmacology, University of Cambridge, Cambridge, United Kingdom; 2 University of Cambridge Metabolic Research Laboratories, Institute of Metabolic Science, Addenbrooke’s Hospital, Cambridge, United Kingdom; 3 Department of Neurology, School of Medicine, Keio University, Shinjuku-ku, Tokyo, Japan; Montreal Diabetes Research Center, Canada

## Abstract

The endoplasmic reticulum localised protein seipin, encoded by the gene *Berardinelli–Seip congenital lipodystrophy type 2 (BSCL2),* serves a critical but poorly defined function in the physiology of both adipose and neural tissue. In humans, *BSCL2* loss-of-function mutations cause a severe form of lipodystrophy, whilst a distinct set of gain-of-toxic-function mutations are associated with a heterogeneous group of neuropathies. However, despite the importance of seipin dysfunction to the pathophysiology of these conditions, little is known about its physiological role in adipocytes or neurons. *BSCL2* mRNA has previously been identified in human and mouse brain, yet no definitive assessment of its expression has been undertaken. Here we comprehensively characterised the neuroanatomical distribution of mouse *Bscl2* using complementary in situ hybridisation histochemistry and immunohistochemistry techniques. Whilst *Bscl2* was broadly expressed throughout the rostral-caudal extent of the mouse brain, it exhibited a discrete neuroanatomical profile. *Bscl2* was most abundantly expressed in the hypothalamus and in particular regions associated with the regulation of energy balance including, the paraventricular, ventromedial, arcuate and dorsomedial nuclei. *Bscl2* expression was also identified within the brainstem dorsal vagal complex, which together with the paraventricular nucleus of the hypothalamus represented the site of highest expression. Further neurochemical profiling of these two nuclei revealed *Bscl2*/seipin expression within energy balance related neuronal populations. Specifically, seipin was detected in oxytocin neurons of the paraventricular nucleus of the hypothalamus and in catecholamine neurons of the dorsal vagal complex. These data raise the possibility that in addition to its role in adipose tissue development, seipin may also be involved in the central regulation of energy balance.

## Introduction

The resident endoplasmic reticulum protein, seipin, encoded by the *BSCL2* gene, has been implicated in both metabolic and neurological disease. These distinct and non-overlapping conditions appear to arise as a consequence of loss- and gain-of-function *BSCL2* mutations respectively, yet specific cellular functions for seipin remain poorly defined [1]–[4].

*BSCL2* was originally identified in a genetic screen of patients suffering from a severe syndrome of congenital generalised lipodystrophy (Berardinelli–Seip congenital lipodystrophy type 2) with autosomal recessive inheritance [5]. In these cases a range of point, premature stop, deletion or frameshift mutations in *BSCL2* are likely to cause a loss of seipin function and lead to an almost complete absence of adipose tissue [5]–[8]. Such congenital lipodystrophy results in the development of a complex metabolic syndrome characterised by severe insulin resistance, hyperlipidaemia and ectopic lipid storage [4]. Several laboratories have now demonstrated critical roles for seipin in adipocyte differentiation and adipose tissue development, implying that seipin disruption causes lipodystrophy largely through a cell-autonomous defect of adipogenesis [9]–[12]. A distinct set of pathogenic gain-of-toxic function mutations in *BSCL2* have been shown to cause a clinically heterogeneous class of autosomal dominant motor neuropathy syndromes, collectively termed the seipinopathies [3], [13]. *BSCL2* point mutations that affect an N-glycosylation site at amino acid 88 (N88S or S90L) result in aggregation of the mutant seipin protein and the proposed pathogenic activation of ER stress pathways [3], [13], [14]. These seipinopathies exhibit broad phenotypic variance across patients but are ultimately defined by upper, lower and/or peripheral motor neuron disturbance but no apparent metabolic symptoms. Importantly, murine modelling of *BSCL2* pathologies reveals significant functional conservation between humans and mice, such that whilst *Bscl2* ablation leads to lipodystrophy [15] the overexpression of an N88S mutant transgene promotes progressive motor deficits [16].

The involvement of *BSCL2* in lipodystrophy and neuropathy is associated with its expression in adipocytes and spinal cord motor neurons respectively. In addition, the reported roles of seipin in lipid droplet morphology and/or biogenesis in multiple cell types and model organisms also indicate roles in normal cellular function [1], [9], [11], [12], [17], [18]. In this regard, the presence of *BSCL2* expression in a range of other human tissue types including brain, liver, kidney, pancreas and testis also warrants further investigation [13].

Here we provide the first comprehensive neuroanatomical analysis of mouse brain *Bscl2* expression. We report that *Bscl2* is expressed in numerous specific structures throughout the brain. We find that *Bscl2* mRNA exhibits a highly defined neuroanatomical profile and that its expression is strongly associated with nuclei implicated in the regulation of energy balance.

## Materials and Methods

### Animals

Male C57BL/6 or mice on a C57BL/6 background expressing enhanced green fluorescent protein (EGFP) under the control of POMC regulatory elements (POMC-EGFP mice; generous gift from Prof. Richard Simerly, University of Southern California and Prof. Malcolm Low, University of Michigan [19]) between the ages of 2–4 months were provided with standard laboratory chow and water *ad libitum* and maintained in a light (12 h on/12 h off) and temperature controlled environment (21.5–22.5°C). All experiments were carried out in accordance with the U.K. Animals (Scientific Procedures) Act 1986, with appropriate ethical approval.

### Tissue Preparation

Mice were deeply anesthetised with pentobarbitone (50 mg/kg i.p.) and transcardially perfused with diethylpyrocarbonate (DEPC)-treated phosphate buffered saline (PBS) followed by 10% neutral buffered formalin (Sigma). Following extraction, brains were post fixed overnight in 10% neutral buffered formalin and then transferred to 20% sucrose for cryoprotection. Brains were sectioned on a freezing sliding microtome at 25 µm and collected in five equal series of adjacent tissue.

### *Bscl2* Riboprobe Synthesis

A *Bscl2* specific riboprobe complementary to exon 7–12 of the mouse coding region (NM_001136064) was generated by PCR using cDNA obtained from mouse brain and the product cloned into a pCR2II-TOPO vector (Invitrogen). The recombinant plasmid was linearized by restriction digest and subject to *in vitro* transcription with a T7 (antisense) or SP6 (sense) RNA polymerase in the presence of ^35^S-labelled UTP (for radioactive in situ hybridisation) or digoxigenin-labelled UTP (for fluorescent in situ hybridisation histochemistry) according to the manufacturer’s instructions (Ambion).

### Radioactive in situ Hybridisation Histochemistry (ISHH)

Sectioned brain tissue was processed for ISHH as previously described [14]. ^35^S-labeled *Bscl2* riboprobe was diluted to 2×10^7^ cpm ml^−1^ in a hybridization buffer composed of 50% formamide, 20 mM Tris-HCl pH 7.5, 0.02% sheared single-stranded DNA (Sigma), 0.1% total yeast RNA (Sigma), 0.01% yeast tRNA (Gibco), 20% dextran sulphate, 0.3 M NaCl, 2 mM EDTA pH 8.0, Denhardt’s solution (Sigma), 100 mM DTT, 0.2% SDS and 0.2% sodium thiosulphate (Sigma). Brain sections were mounted onto superfrost slides and allowed to air dry. Sections were fixed in 4% formaldehyde in DEPC-treated PBS for 20 min at 4°C, dehydrated in ascending concentrations of ethanol, cleared in xylene for 15 min, rehydrated in descending concentrations of ethanol and permeabilised by heating in sodium citrate buffer (95–100°C, pH 6.0), before being dehydrated in ascending concentrations of ethanol, and air-dried. Hybridization solution (containing radiolabelled riboprobe) and a coverslip were applied to each slide, and sections were incubated for 16 hrs at 57°C. Coverslips were then removed, and slides were washed with 2× sodium chloride/sodium citrate buffer (SSC). Sections were incubated in 0.002% RNase A (Qiagen) for 30 min, followed by sequential washes in decreasing concentrations of SSC. Sections were dehydrated in ascending concentrations of ethanol with 0.3 M ammonium acetate (NH_4_OAc) followed by 100% ethanol. Slides were air-dried and placed in X-ray film cassettes with BMR-2 film (Kodak) for 72 hrs. Films were developed on an OPTIMAX X-ray film processor (Protec). Slides were subsequently dipped in photographic emulsion (GE Healthcare) and stored at 4°C for 2 wks before being developed in D-19 developer and fixer (Kodak). Slides were imaged under darkfield microscopy.

### Dual-fluorescent in situ Hybridisation and Immunofluorescent Histochemistry

A digoxigenin-labelled *Bscl2* riboprobe was diluted 1/200 in the hybridisation buffer described above. Brain tissue mounted onto superfrost slides was fixed in 4% formaldehyde in DEPC-treated PBS for 20 min at 4°C, dehydrated in ascending concentrations of ethanol, cleared in xylene for 15 min, rehydrated in descending concentrations of ethanol and permeabilised by heating in sodium citrate buffer (95–100°C, pH 6.0), before being dehydrated in ascending concentrations of ethanol, and air-dried. Hybridization solution (containing digoxigenin-labelled *Bscl2* riboprobe) and a coverslip were applied to each slide, and sections were incubated for 16 hrs at 57°C. Tissue was then processed for fluorescent in situ hybridisation. Briefly, following washing in decreasing concentrations of SSC, slides were rinsed in TNT buffer (0.1 M TRIS-HCL pH 7.5, 0.15 M NaCl, 0.3% Triton-X-100) and blocked for 30 min in TNB buffer (0.1 M TRIS-HCL pH 7.5, 0.15 M NaCl, 0.5% blocking reagent) at room temperature in a humidified chamber. Sections were then incubated in a horseradish peroxidase conjugated anti-digoxigenin antibody (Roche) diluted 1/100 in TNB buffer. Following a series of TNT washes slides were treated with tyramide signal amplification (TSA) biotin plus kit, as per manufactures instructions (PerkinElmer). Probe detection was achieved using an anti-streptavidin 594 antibody diluted 1/250 in TNB buffer at room temperature for 30 min. After extensive washing in PBS slides were processed for immunofluorescent histochemical detection of seipin. Slides were incubated in blocking buffer containing 0.5% BSA, 0.5% Triton-X 100 in PBS for 1 hr at room temperature and then incubated overnight at room temperature in blocking buffer containing rabbit anti-seipin antibody (1/1000; [20]). Sections were subsequently washed in PBS followed by incubation with a donkey anti-rabbit 488 antibody (1/1000 in blocking buffer, Alexa Fluor, Molecular Probes) for 1 hr at room temperature. Slides were washed in PBS and cover-slipped in an aqueous mounting medium (Vectastain; Vector Laboratories).

### Immunofluorescent Histochemistry

Free floating sections were washed in PBS, incubated in blocking buffer (0.5% BSA, 0.5% Triton-X 100 in PBS) for 1 hr and then incubated overnight in blocking buffer containing rabbit anti-seipin antibody (1/1000). Sections were subsequently washed in PBS followed by incubation with a donkey anti-rabbit 568 antibody (1/1000 in blocking buffer, Alexa Fluor, Molecular Probes) for 1 hr. Sections were then washed in PBS, incubated in blocking buffer and incubated overnight with one of the following primary antibodies: rabbit anti-oxytocin (1/1000, Phoenix Pharmaceuticals), mouse anti-tyrosine hydroxylase (1/1000, Chemicon) or goat anti-green fluorescent protein (1/1000, Abcam). Sections were subsequently washed in PBS followed by 1 hr incubation in blocking buffer containing appropriate secondary antibody (1/1000; 488 Alexa Fluor, Molecular Probes). Sections were rinsed in PBS, mounted onto glass microscope slides and cover-slipped in an aqueous mounting medium (Vectastain; Vector Laboratories).

### Imaging and Analysis

Slides were viewed on a Zeiss Axioskop 2 fluorescent microscope and images captured using Zeiss AxioCam HRc digital camera and AxioVision software. Fluorescent images were merged using Adobe Photoshop CS3. Double labelled cells were recorded if both fluorescent signals were present in the same cell and each alone clearly defined a cellular shape.

## Results

### Neuroanatomical Distribution of Mouse *Bscl2* mRNA

**Figure 1:**
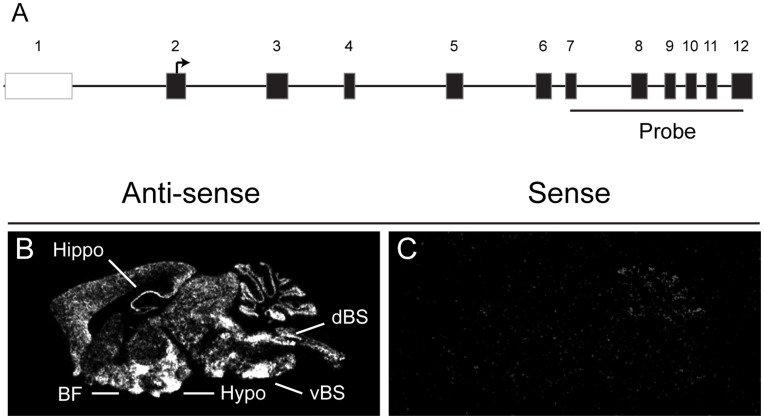
Expression of *Bscl2* mRNA in adult mouse brain. Autoradiographical visualisation of *Bscl2* mRNA in saggital section of the adult mouse brain using radioactive in situ hybridisation histochemistry. (**A**) Diagrammatic representation of the mouse *Bscl2* locus detailing the location of the ISHH riboprobe (not to scale). (**B**) Endogenous *Bscl2* expression as detected by a specific antisense riboprobe revealed strong expression within the basal forebrain (BF), hippocampus (Hippo), hypothalamus (Hypo), dorsal brainstem (dBS) and ventral brainstem (vBS). (**C**) Corresponding *Bscl2* sense riboprobe control.

**Figure 2:**
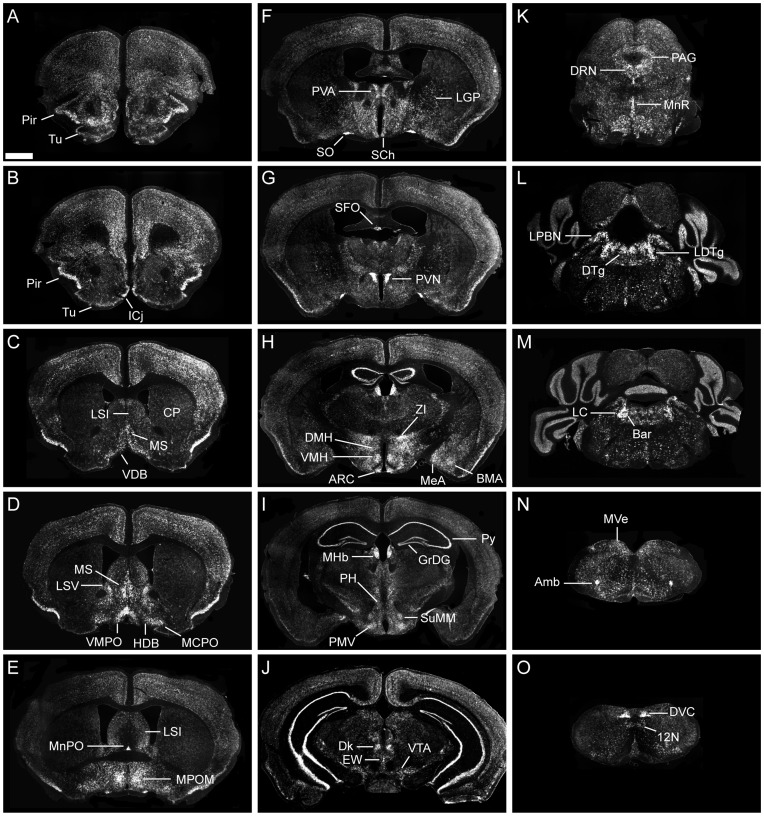
Neuroanatomical characterisation of *Bscl2* mRNA in adult mouse brain. Radioactive in situ hybridisation histochemistry analysis of *Bscl2* mRNA distribution in coronal section across the rostral-caudal extent of adult mouse brain. Endogenous *Bscl2* expression was detected throughout the brain, for full characterisation see Table 1. (**A–O**) ^35^S-labelled *Bscl2* expression was detected in the piriform cortex (Pir), olfactory tubercle (Tu), islands of Calleja (ICj), caudate putamen (CP) lateral septal nucleus intermediate part (LSI), medial septal nucleus (MS), nucleus of the vertical limb of the diagonal band (VDB), lateral septal nucleus ventral part (LSV), nucleus of the horizontal limb of the diagonal band (HDB), magnocellular preoptic nucleus (MCPO), ventromedial preoptic nucleus (VMPO), median preoptic nucleus (MnPO), medial preoptic nucleus medial part (MPOM), paraventricular thalamic nucleus (PVA), lateral globus pallidus (LGP), supraoptic nucleus (SO), suprachiasmatic nucleus (SCh), subfornical organ (SFO), paraventricular nucleus of the hypothalamus (PVN), zona incerta (ZI), dorsomedial nucleus of the hypothalamus (DMH), ventromedial nucleus of the hypothalamus (VMH), arcuate nucleus of the hypothalamus (ARC), basomedial amygdaloid nucleus (BMA), medial amygdaloid nucleus (MeA), medial habenular (MHb), pyramidal cell layer of the hippocampus (py), granular layer of the dentate gyrus (GrDG), posterior hypothalamus (PH), supramammilliary nucleus medial part (SuMM), premammillary nucleus ventral part (PMV), nucleus of Darkschewitsch (Dk), Edinger-Westphal nucleus (EW), ventral tegmental area (VTA), dorsal raphe nucleus (DRN), periaqueductal grey (PAG), median raphe nucleus (MnR), lateral parabrachial nucleus (LPBN), dorsal tegmental nucleus (DTg), laterodorsal tegmental nucleus (LDTg), locus coeruleus (LC), Barrington’s nucleus (Bar), medial vestibular nucleus (MVe), ambiguous nucleus (Amb), dorsal vagal complex (DVC), hypoglossal nucleus (12N). Scale bar in (A) represents 1 mm and applies to all other images.

Expression of brain *Bscl2* mRNA across the rostral-caudal extent of the mouse brain was investigated using a *Bscl2* specific riboprobe (corresponding to exon 7–12 of the mouse *Bscl2* coding region, Fig. 1A–C) and radioactive ISHH. Gross saggital section analysis revealed extensive but discrete *Bscl2* expression throughout the mouse neuraxis with robust expression detected in the basal forebrain at the level of the preoptic area, the hypothalamus and dorsal and ventral aspects of the brainstem (Fig. 1B). More neuroanatomically refined assessment of *Bscl2* mRNA distribution demonstrated a broad but region specific profile (Fig. 2 and Table 1).

**Table 1:** Distribution of *Bscl2* mRNA in the mouse central nervous system.

**Cerebral cortex**	
Cingulate cortex	+
Motor cortex	+
Primary somatosensory cortex	+
Visual cortex	+
Orbital cortex	+
Indusium griseum	++
Olfactory nucleus	++
Olfactory turbcle	+
Piriform cortex	++++
**Striatum**	
Globus pallidus, lateral	SC (++)
Globus pallidus, medial	SC (++)
Ventral pallidum	+
**Hippocampus and septum**	
Dentate gyrus, granular layer	++
Island of Calleja	++
Lateral septal nucleus, ventral	++
Lateral septal nucleus, dorsal	+
Medial septal nucleus	+++
Nucleus of the limb diagonal band, ventral	++
Nucleus of the limb diagonal band, horizontal	++
Magnocellular preoptic nucleus	++
Pyramidal cell layer of hippocampus	++++
Septohippocampal nucleus	++
Subfornical organ	+++
Bed nucleus of the stria terminalis, medial div	+
Bed nucleus of the stria terminalis, lateral div	+
**Amygdala**	
Basolateral amygdaloid nucleus, anterior	+++
Central amygdaloid nucleus	++
Medial amygdaloid nucleus, posterodorsal	+++
Medial amygdaloid nucleus, posteroventral	++
**Thalamus**	
Anterior thalamic nucleus	+
Central medial thalamic nucleus	++
Paraventricular thalamic nucleus anterior	++
Lateral habenular	+
Medial habenular	++++
Subparafascicular thalamic nucleus	SC (++)
Reticular thalamic nucleus	+++
Reuniens nucleus	++
Zona incerta	++++
Subincertal nucleus	++++
**Hypothalamus**	
Acruate nucleus	++++
Anterior hypothalamic nucleus	++
Dorsomedial nucleus, mediodorsal	+++
Dorsomedial nucleus, central	+++
Dorsomedial nuzcleus, medioventral	+++
Lateral hypothalamic area	SC (++)
Lateral mammillary nucleus	++
Lateral preoptic area	+++
Medial mammillary nucleus	+
Median preoptic nucleus	++++
Median preoptic nucleus, medial	++++
Median preoptic nucleus, lateral	++++
Medial preoptic area	+++
Paraventricular nucleus, ventral	+++++
Paraventricular nucleus, lateral magnocellular	+++++
Paraventricular nucleus, medial magnocellular	+++++
Paraventricular nucleus, anterior parvicellular	SC (++)
Paraventricular nucleus, medial parvicellular	SC (+)
Paraventricular nucleus, posterior	+++
Posterior hypothalamus	+++
Premammillary nucleus, dorsal	++
Premammillary nucleus, ventral	+++
Subthalamic nucleus	SC (+)
Suprachiasmatic nucleus	+++
Supraoptic nucleus	+++++
Supramammillary nucleus, medial	++
Ventromedial nucleus, central	++
Ventromedial nucleus, dorsomedial	+++
Ventromedial nucleus, ventrolateral	+++
Ventromedial preoptic nucleus	+++
**Midbrain and Pons**	
Barrington’s nucleus	+++
Caudal linear nucleus of raphe	++
Central gray of the pons	+
Dorsal raphe nucleus	+++
Dorsal tegmental area	+++
Edinger-Westphal nucleus	+++++
Lateral parabrachial nucleus	++
Lateral reticular nucleus	
Laterodorsal tegmental area	+++
Laterodorsal tegmental nucleus	++
Locus coeruleus	++++
Medial parabrachial nucleus	SC (+)
Median Raphe	+++
Nucleus of Darkschewitch	+++
Periaqueductal Grey	+
Parabigeminal nucleus	+
Pontine nuclei	+++
Pontine nucleus, oral part	SC (++)
Pontine reticular nucleus, caudal part	SC (+)
Principal sensory trigeminal nucleus	SC (+)
Red nucleus	SC (++)
Reticulotegmental area	SC (++)
Reticulotegmental nucleus of the pons	++
Substantia nigra pars compacta	++
Superior colliculus	+
Ventral tegmental area	++
Ventral tegmental nucleus	++
**Cerebellum**	
Granular layer	+
**Medulla**	
Abducens nucleus	SC (++)
Ambiguous nucleus	+++++
Dorsal motor nucleus of the vagus	+++
Facial nucleus	SC (++)
Gigantocellular reticular nucleus	SC (+)
Hypoglossal nucleus	+++
Medial vestibular nucleus	++
Nucleus of the solitary tract, medial	+++
Nucleus of the solitary tract, lateral	+++
Raphe Magnus	+ SC
Raphe Obscurus	+ SC
Raphe Pallidus	+ SC

Qualitative assessment of *Bscl2* mRNA expression across the rostral-caudal extent of the adult mouse brain, as indicated by radioactive in situ hybridisation histochemistry. Levels of expression: +, very low; ++, low; +++, moderate; ++++, high; +++++, very high; SC (+), scattered cells of low expression; SC (++) scattered cells of high expression.

The cerebral cortex exhibited a consistent but relatively low level of *Bscl2* expression in all neuroanatomical domains. This expression was moderately higher within the olfactory nuclei of the cortex but robustly elevated in the piriform cortex (Pir; Fig. 2A). Within the basal ganglia, expression was significantly lower; only scattered cells were evident within the globus pallidus (although the level of cellular expression was noticeably robust) and no detectable expression was observed within the caudate putamen (Fig. 2C). At the midline, *Bscl2* mRNA expression was highest in medial septal nucleus (MS; Fig. 2C, D) and extended ventrolaterally into the nucleus of the diagonal band (vertical and horizontal) and magnocellular preoptic area (MCPO; Fig. 2D). Expression within the lateral septal nucleus was predominantly confined to the ventral domain (LSV; Fig. 2D). Low level expression was found in the bed nucleus of the stria terminalis but no significant signal was detected in the nucleus accumbens.

*Bscl2* expression within the thalamus was generally low, although the anterior paraventricular nucleus of the thalamus (PVA; Fig. 2F) exhibited a significant level of *Bscl2* mRNA, with weaker expression detected at the posterior extent of the nucleus. Expression was also evident within the central medial thalamic nucleus, reticular thalamic nucleus and reuniens nucleus. Very high levels of *Bscl2* mRNA were detected in the medial habenular nucleus (MHb; Fig. 2I) and, at the thalamic/hypothalamic boundary, within the zona incerta (ZI; Fig. 2H) and subincertal nucleus. Expression within the hippocampus (Fig. 2I) was restricted to the granular layer of the dentate gyrus (GrDG) and the pyramidal cell layer (Py) and strikingly high in both structures.

**Figure 3:**
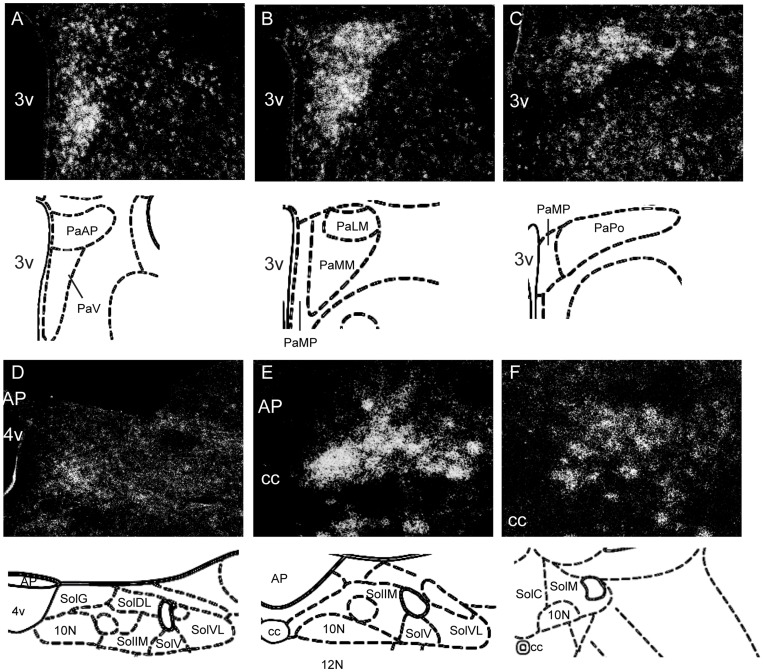
*Bscl2* mRNA distribution within the PVN and DVC. Radioactive in situ hybridisation histochemistry analysis of *Bscl2* mRNA distribution in coronal section across three rostral-to-caudal levels of adult PVN and DVC. (**A–C**) ^35^S-labelled *Bscl2* mRNA expression in the PVN demonstrating robust labelling in the ventral (A), medial magnocellular (B), lateral magnocellular (B, C) and posterior domains (C). Scattered *Bscl2* labelled cells were expressed in the anterior and medial (B, C) parvicellular portion. (**D–F**) ^35^S-labelled *Bslc2* mRNA expression in the DVC was highest within the 10N at the level of the area postrema. Within the NTS the preponderance of *Bscl2* mRNA was localised to the medial and ventral domains (E, F). No expression was detected in the area postrema (E). 4v, fourth ventricle; 10N, dorsal motor nucleus of the vagus; 12N, hypoglossal nucleus; AP, area postrema; cc, central canal; PaAP, PVN anterior parvicellular; PaLM, PVN lateral magnocellular; PaMM, PVN medial magnocellular; PaMP, PVN medial parvicellular; PaPo, PVN posterior; PaV, PVN ventral; SolC, NTS commissural; SolDL, NTS dorsolateral; SolG, NTS gelatinous; SolIM, NTS intermediate; SolM, NTS medial; SolV, NTS ventral; SolVL, NTS ventrolateral.

The hypothalamus represented the highest concentration of *Bscl2* mRNA within the adult mouse brain (Fig. 2G–I). At the anterior extreme, *Bscl2* was robustly expressed within preoptic area (Fig. 2D, E); in particular the median preoptic nucleus (all domains) and to a lesser extent the ventromedial preoptic nucleus (VMPO) and median preoptic area (MPA). In general, *Bscl2* expression extended uniformly though the extent of each relevant nucleus. The paraventricular nucleus of the hypothalamus (PVN; Fig. 2G) was the most abundant site of *Bscl2* expression within the brain. *Bscl2* mRNA within the PVN was robustly expressed in the ventral (Fig. 3A), magnocellular (medial and lateral portions; Fig. 3B) and posterior domains (Fig. 3C). Scattered expression was evident within the anterior parvicellular (Fig. 3A) nucleus and to a lesser extent the medial parvicellular region (Fig. 3B). The supraoptic nucleus (SO; Fig. 2F) also demonstrated very high levels of *Bscl2* mRNA. Lower levels were detected within the anterior, dorsomedial (DMH), arcuate (ARC) and posterior hypothalamic (PH) nuclei (,2H). The lateral hypothalamus (LH) contained only scattered cells. The ventromedial hypothalamus (VMN) exhibited regional variance in *Bscl2* expression with the central portion demonstrating lower levels than that observed in the dorsomedial and ventrolateral domains (Fig. 2H). Within more caudal regions, *Bscl2* was detected in the ventral and dorsal premammillary nucleus and the central supramammillary nucleus (SuMM; Fig. 2I).

Within the midbrain, moderate *Bscl2* expression was apparent in the subsantia nigra pars compacta (SnC) and adjacent ventral tegmental area (VTA; Fig. 2J), but not the subsantia nigra pars reticulata. At the level of the cerebral aqueduct, two sites of robust expression were identified as the Edinger-Westphal (EW) nucleus and the nucleus of Darkschewitch (Dk; Fig. 2J). Caudal to this, low level expression was detected in all regions of the periaqueductal grey (PAG) and the superior colliculus. The dorsal raphe nucleus (DRN) exhibited the greatest level of *Bscl2* mRNA within the raphe nuclei, with the dorsal portion containing the highest expression (Fig. 2K). The median (MnR) and caudal linear raphe demonstrated more uniform but moderate expression (Fig. 2K). The brainstem raphe nuclei (magnus, obscurus and pallidus) were typified by scattered *Bscl2* containing cells. Within the pons, robust expression was detected in the locus coeruleus (LC; Fig. 2M) and to a lesser extent the lateral parabrachial nucleus (LPBN; Fig. 2L), laterodorsal (LDTg) and dorsal tegmental nuclei (DTg; Fig. 2L). Only the granular layer of the cerebellum was positive for *Bscl2* expression.

Brainstem *Bscl2* expression was evident within a number of cranial nuclei, specifically, the abducens nucleus (6N), facial nucleus (7N) and hypoglossal nucleus (12N). Very high expression was detected in the ambiguous nucleus (Fig. 2N). Moderate expression was detected in the medial vestibular nucleus (MVe) lateral to the fourth ventricle (Fig. 2N). The dorsal vagal complex (DVC), comprised of the nucleus of the solitary tract (NTS), the dorsal motor nucleus of the vagus (10N) and area postrema, exhibited robust expression that was highest in the 10N at the subpostremal level (Fig. 2O and Fig. 3). No expression was detected in the area postrema, whilst the NTS exhibited robust *Bscl2* signal within the medial and lateral domains (Fig. 3E and F).

### Seipin Immunoreactivity within the PVN and DVC

**Figure 4:**
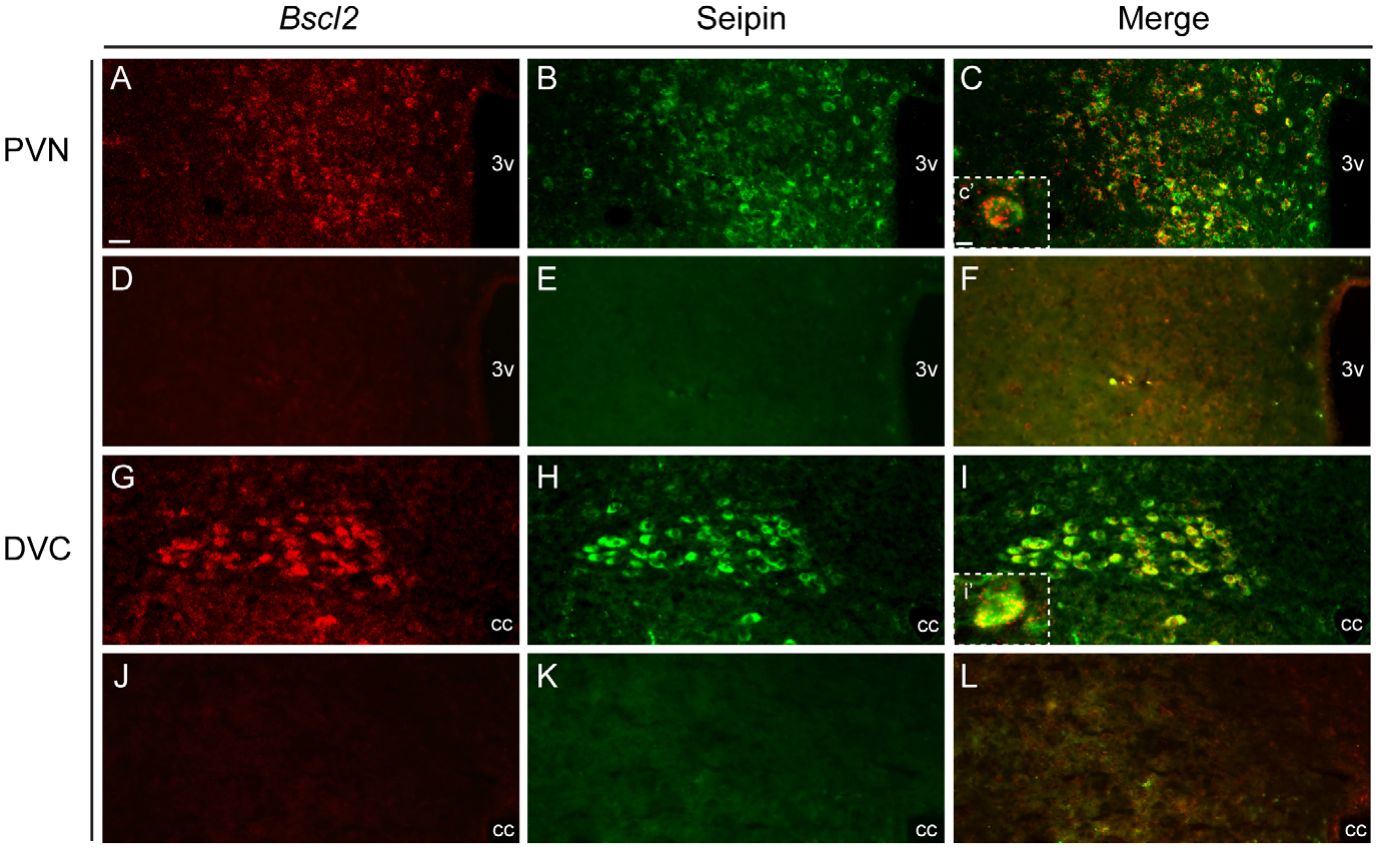
Validation of seipin antibody in the PVN and DVC of adult mouse. Dual *Bscl2* fluorescent in situ hybridisation and seipin immunohistochemistry. (**A–C**) Co-localisation of *Bscl2* mRNA and seipin in the magnocellular domain of the paraventricular nucleus of the hypothalamus (PVN) and (**G–I**) dorsal vagal complex (DVC). Co-localisation denoted by yellow colouring in panels C and I. Negative controls for fluorescent in situ hybridisation and immunohistochemical protocols in the PVN (**D–F**) and DVC (**J–L**) revealed no non-specific staining. Inserts (**C′**) and (**I′**) show high magnification images of cellular co-localisation. Scale bar in (A) represents 25 µm and applies to figures A**–**L; scale bar in (C′) represents 10 µm and applies to I′.3v, third ventricle; cc, central canal.

The abundant expression of *Bscl2* within energy balance associated nuclei of the brain, together with its established involvement in peripheral metabolism, prompted further investigation of two central sites of robust *Bscl2* expression, the PVN and DVC. Validation of the seipin antibody (SCT14) for subsequent neurochemical profiling was undertaken using fluorescent ISHH for *Bscl2* combined with fluorescent immunohistochemistry for seipin (Fig. 4). Dual labelling for *Bscl2* and seipin in the PVN was evident in the vast majority of cells at all levels of the nucleus (Fig. 4A–C) and was absent in negative controls (Fig. 4D–F). Similar results were obtained within the DVC (Fig. 4G–L).

### Neurochemical Profiling of Seipin Expressing Cells in the PVN and DVC

**Figure 5:**
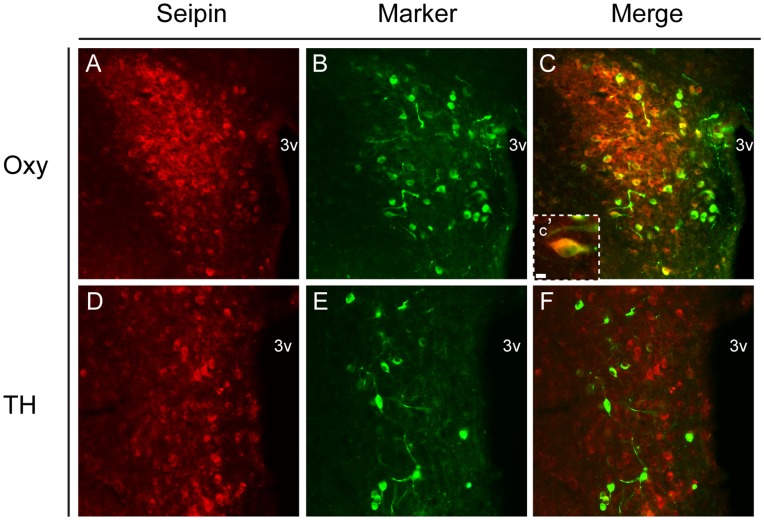
Neurochemical profile of seipin positive neurons in the PVN of adult mouse. Dual immunofluorescent colocalisation of seipin and (**A–C**) oxytocin (Oxy) and (**D–E**) tyrosine hydroxylase (TH) in the paraventricular nucleus of the hypothalamus (PVN). Colocalisation with oxytocin was evident within PVN (as denoted by yellow colouring in panel C and C′). No colocalisation was observed with TH. Scale bar in (A) represents 25 µm and applies to figures A–E; scale bar in (C′) represents 10 µm. 3v, third ventricle.

**Figure 6:**
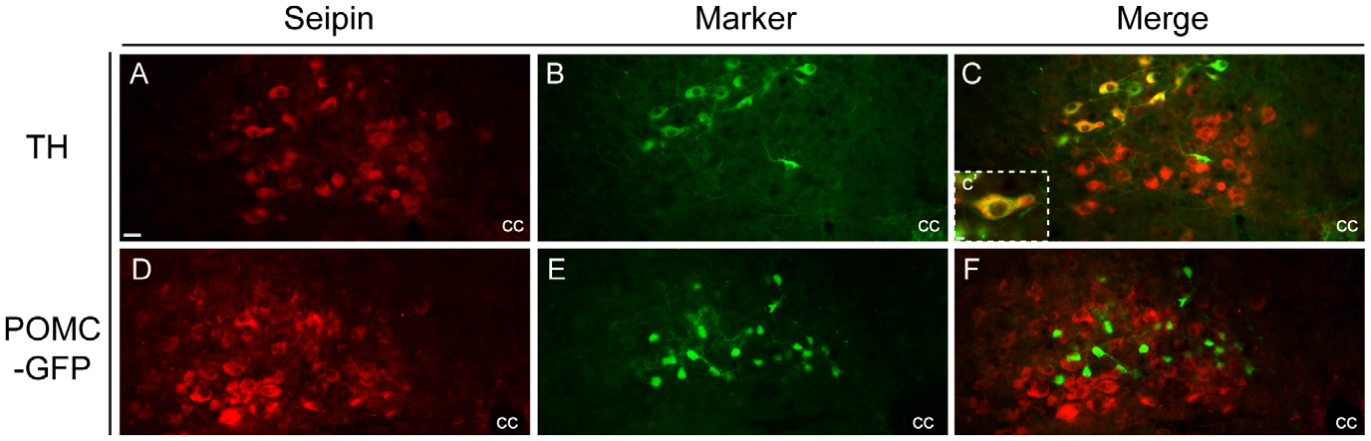
Neurochemical profile of seipin positive neurons in the DVC of adult mouse. Dual immunofluorescent co-localisation of seipin and (**A–C**) tyrosine hydroxylase (TH) and (**D–E**) green fluorescent protein under the control of the pro-opiomelanocortin promoter (POMC-EGFP) in the nucleus of the solitary tract (NTS). Co-localisation with TH was evident within NTS (as denoted by yellow colouring in panel C and C′). No co-localisation was observed with GFP. Scale bar in (A) represents 25 µm and applies to figures A-E; scale bar in (C′) represents 10 µm. 3v, third ventricle. cc, central canal.

The cellular heterogeneity of the PVN and DVC raised questions as to the neurochemical nature of seipin expressing cells. Dual immunofluorescent co-localisation of seipin with markers for PVN neuronal populations indicated that oxytocin cells of both the parvicellular and magnocellular portions were positive for seipin expression (Fig. 5A–C). However, catecholamine neurons (as labelled by tyrosine hydroxylase (TH) immunoreactivity) of the ventral PVN were seipin negative (Fig. 5D–F). In the DVC, seipin was co-expressed with TH (Fig. 6A–C), but not pro-opiomelanocortin (POMC) neurons (as labelled by GFP in a transgenic POMC-EGFP line) (Fig. 6D–F). In contrast, a subpopulation of hypothalamic POMC neurons did co-express seipin (data not shown).

## Discussion

Despite the lack of a precisely defined cellular function, appropriate expression of wild-type *Bscl2* is critical to the development and physiology of both adipose and neural tissue. Unsurprisingly, the role of seipin in the manifestation of lipodystrophy and motor neuropathy has dominated much of the current research. However, in light of such a strong pathological association, evidence of *Bscl2* expression in numerous other tissues also warrants further investigation.

Previous analysis of central *BSCL2* expression in human brain utilised northern blotting of macro-dissected tissue homogenates. These findings indicated seemingly ubiquitous *BSCL2* expression in the cerebellum, cerebral cortex, frontal, temporal and occipital lobes, and to a lesser extent the medulla and caudate putamen [13]. No detailed analysis of *Bscl2* mRNA distribution in mouse brain has been reported, although its presence in whole brain samples has been demonstrated by RT-PCR [13]. Our combined utilisation of in situ hybridisation histochemistry (for *Bscl2* mRNA) and immunohistochemistry (for seipin protein) now provides insight into the specific neuroanatomical distribution of mouse *Bscl2*/seipin.

The breadth of *Bscl2* expression observed in the mouse is consistent with that previously reported of the human brain. Specifically we identified mRNA expression at all levels of the brain, with a more refined profile evident within the hypothalamus and brainstem. A first set of seipin-expressing regions directly concerns motor control, which is in keeping with the major phenotypic trait of seipinopathies. Interestingly, contrary to human data we find no evidence of *Bscl2* expression in the caudate putamen [13], although scattered expression was apparent in the globus pallidus. The expression of *Bscl2* within the motor cortex may be of relevance to Silver Syndrome, a seipinopathy characterised in part by upper motor neuropathy [3]. Indeed, motor impairment in N88S mutant mice is correlated with mutant seipin aggregates in the motor cortex neurons [16]. Furthermore, *Bscl2* is robustly expressed in the ambiguous nucleus (XI cranial nerve) of the rostral medulla, the source of efferent motor fibres to the muscles groups of the shoulder and neck, and may be associated with the upper body weakness (in particular the shoulders) observed in Silver Syndrome patients [3]. The presentation of ocular defects in some seipinopathies [21] seems unlikely to be associated with disruption of cranial nerve innervation of the eye, as *Bscl2* expression was not detected in the optic (2N), occulomotor (3N) or trochlear (4N) nuclei.

*BSCL2* loss-of-function mutations underlie the congenital generalised lipodystrophy BSCL2, characterised by a near complete absence of adipose tissue and associated metabolic dysregulation. Some BSCL2 patients have also been reported to display cognitive impairment [6]. Although this aspect of the phenotype is less clear than that affecting adipose tissue, it remains possible that central *BSCL2* expression is of importance to neurodevelopment and cognition.

Robust *Bslc2* expression was also detected in neural structures concerned with the regulation of energy balance, namely the hypothalamus and brainstem (DVC). The cross-talk between the brain and adipose tissue is clearly central to the regulation of body weight with both hormonal and neurological connections between these two tissues providing bidirectional modulation of their respective functions [22], [23]. For example, the level of the adipostatic hormone leptin rises with fat accrual and acts directly within the brain to communicate long-term nutritional fitness and short term appetitive state, leading to centrally mediated activation of appropriate physiological and behavioural responses [24]. Equally, sympathetic innervation of fat enables central control of lipid metabolism and thus energy storage [25]. Interestingly, a number of genes highly expressed in the adipocyte are also found in the brain, and particularly the hypothalamus, suggesting a level of functional convergence. The hypothalamus represents the most abundant site of *Bscl2* mRNA in the mouse brain, implying a potential involvement in homeostatic aspects of physiology and behaviour. In this regard, it is of note that robust *Bscl2* expression was detected in a number of hypothalamic nuclei intimately associated with the regulation of energy homeostasis and body weight, specifically, the paraventricular, arcuate, ventromedial and dorsomedial nuclei. *Bscl2* expression was also identified within the DVC, a brainstem nucleus comprised of specific sub-nuclei with an implicit involvement in the energy balance, including the NTS which serves as a integrative node through which numerous peripheral appetitive cues are routed and the 10N that provides direct neural innervation and regulation of peripheral tissues, (including adipose tissue, liver and intestine/stomach) and is a target for the actions of peripheral appetitive cues such as leptin and cholecytokinin [26]. Moreover, the PVN and DVC represent the sites of highest *Bscl2* expression in the mouse brain.

Neurochemical profiling of seipin-immunoreactive neurons within the PVN and DVC identified its expression in oxytocin and catecholaminergic neurons, respectively. Both these populations are associated with the regulation of energy balance [27], [28]. Seipin expression within the PVN was predominantly localised to the magnocellular domains, with only scattered cells identified in the parvicellular regions. Oxytocin neurons in the mouse exhibit a similar neuroanatomical profile such that they are predominantly localised to the magnocellular PVN with far fewer cells evident in the parvicellular PVN. Our colocalisation studies indicate that in both regions seipin colocalised with oxytocin. Although studies in rats have identified a functional divergence of magnocellular versus parvicellular PVN oxytocin neurons, with the former being associated with the neuroendocrine control of lactation, parturition and osmoregularity and the latter implicated in the regulation of feeding, the situation in the mouse is less clear. Importantly, there is a distinct paucity of oxytocin expressing neurons within the parvicellular region of mice as compared to the rat, as well as significant differences in the neuroarchitecture of the mouse and rat PVN [29], [30]. Thus in the mouse the function divergence of oxytocin neuron populations (neuroendocrine versus CNS projecting) may not be solely defined by their neuroanatomical location. Nevertheless, the importance of oxytocin neurons to the regulation of energy balance in the mouse is well established [27], [31] and provides circumstantial support for involvement of seipin in this function (as well as other aspects of oxytocin regulated physiology). Broad seipin-immunoreactivity within non-oxytocin neurons of the magnocellular PVN illustrates its expression in additional populations of cells that may include corticotrophin-releasing hormone, vasopressin, somatostatin and thyrotropin-releasing peptide. Seipin expression within the DVC was identified in the A2 noradrenergic population of the NTS but not in POMC-EGFP labelled cells. The A2 population is implicated in the homeostatic regulation of cardiovascular reflex and stress. These neurons are also associated with the regulation of energy balance inasmuch as they are reactive to peripheral appetitive cues including amylin [28] and an indirect target for cholecytokinin [32] and ghrelin [33] (via the modulation of glutamatergic vagal afferents). Interestingly, there is a significant degree of reciprocal innervations between the PVN and DVC that includes A2 neuron projection to the PVN [34] and oxytocin neuron projection to the NTS [35]. Whether seipin expression in these cells types is of functional relevance to this neuroanatomical circuit remains to be investigated. That seipin was not identified within catecholaminergic neurons of the PVN or POMC neurons of the NTS (but was evident in POMC-EGFP neurons of the ARC) suggests a degree of functional specificity that remains to be clarified.

Whilst it is at present impossible to attribute these observations with physiological function, the expression of *Bscl2* in both fat and energy balance associated brain regions raises the possibility that seipin may serve to influence energy homeostasis at both the peripheral and central level. Although BSCL2 patients exhibit dramatically increased appetite and increased metabolic rate this most likely reflects the profound lack of leptin due to the paucity of adipose tissue [36]. Seipin deficient mice do not appear to exhibit perturbed feeding behaviour [15] but it is possible that both the age of the mice in the study, the nature of the analysis and/or the severity of this mutant’s pathology may mask an as yet unidentified energy balance phenotype. Interestingly, seipin deficient mice exhibit a significant increase in body temperature despite a 40% reduction in brown adipose tissue (BAT) [15]. Whilst a complete assessment of this phenotype has not yet been undertaken, it is possible that loss of seipin in the brain promotes a centrally mediated increase in BAT metabolic activity.

The role of seipin in adipocyte development appears, in part, to involve an ability to regulate the expression or function of the adipocyte differentiation factor peroxisome proliferator-activated receptor gamma (PPARγ) [10], [37]. Dominant negative PPARγ mutations in humans also result in lipodystrophy [38]. It is of note that both these genes are also expressed in the brain and exhibit overlapping regional expression profiles, including a number of hypothalamic nuclei [39]. This offers the intriguing possibility that seipin might regulate PPARγ expression or function in the brain. Recent studies of neuronal PPARγ have highlighted its critical role in the regulation of energy balance and suggest that the inhibition of central PPARγ expression protects against the hyperphagia and reduced energy expenditure, and thus weight gain, associated with high fat diet induced obesity [40]. Furthermore, whilst speculative, hyperthermia in *Bscl2* knockout mice could be associated with an attenuation of PPARγ function since neuronal specific PPARγ deficient mice exhibit an upregulation of thermogenic gene expression [40]. In light of these observations and the severe metabolic pathology associated with global *Bscl2* deficiency, the generation of a neuron-specific *Bscl2* knockout line will be critical to unravelling any potential contribution of this gene to the central regulation of energy balance.

In summary, our analysis of central *Bscl2* expression reveals a refined and provocative neuroanatomical profile, with the preponderance of expression localised to specific energy balance related nuclei of the hypothalamus and brainstem. In light of the established pathophysiological relevance of adipocyte seipin expression in the regulation of energy storage, these data raise the possibility that neuronal seipin expression may serve an as yet unidentified role in the central regulation of energy homeostasis.

## References

[1] Cartwright BR, Goodman JM (2012) Seipin: from human disease to molecular mechanism. J Lipid Res 53: 1042–1055. 22474068 10.1194/jlr.R023754 PMC3351812

[2] Fei W, Du X, Yang H (2011) Seipin, adipogenesis and lipid droplets. Trends Endocrinol Metab 22: 204–210. 21497513 10.1016/j.tem.2011.02.004

[3] Ito D, Suzuki N (2009) Seipinopathy: a novel endoplasmic reticulum stress-associated disease. Brain 132: 8–15. 18790819 10.1093/brain/awn216

[4] Rochford JJ (2010) Molecular mechanisms controlling human adipose tissue development: insights from monogenic lipodystrophies. Expert Rev Mol Med 12: e24. 20673380 10.1017/S1462399410001547

[5] Magre J, Delepine M, Khallouf E, Gedde-Dahl T Jr, Van Maldergem L, et al (2001) Identification of the gene altered in Berardinelli-Seip congenital lipodystrophy on chromosome 11q13. Nat Genet 28: 365–370. 11479539 10.1038/ng585

[6] Agarwal AK, Simha V, Oral EA, Moran SA, Gorden P, et al (2003) Phenotypic and genetic heterogeneity in congenital generalized lipodystrophy. J Clin Endocrinol Metab 88: 4840–4847. 14557463 10.1210/jc.2003-030855

[7] Simha V, Garg A (2003) Phenotypic heterogeneity in body fat distribution in patients with congenital generalized lipodystrophy caused by mutations in the AGPAT2 or seipin genes. J Clin Endocrinol Metab 88: 5433–5437. 14602785 10.1210/jc.2003-030835

[8] Van Maldergem L, Magre J, Khallouf TE, Gedde-Dahl T Jr, Delepine M, et al (2002) Genotype-phenotype relationships in Berardinelli-Seip congenital lipodystrophy. J Med Genet 39: 722–733. 12362029 10.1136/jmg.39.10.722 PMC1734991

[9] Boutet E, El Mourabit H, Prot M, Nemani M, Khallouf E, et al (2009) Seipin deficiency alters fatty acid Delta9 desaturation and lipid droplet formation in Berardinelli-Seip congenital lipodystrophy. Biochimie 91: 796–803. 19278620 10.1016/j.biochi.2009.01.011

[10] Chen W, Yechoor VK, Chang BH, Li MV, March KL, et al (2009) The human lipodystrophy gene product Berardinelli-Seip congenital lipodystrophy 2/seipin plays a key role in adipocyte differentiation. Endocrinology 150: 4552–4561. 19574402 10.1210/en.2009-0236 PMC2754678

[11] Fei W, Shui G, Gaeta B, Du X, Kuerschner L, et al (2008) Fld1p, a functional homologue of human seipin, regulates the size of lipid droplets in yeast. J Cell Biol 180: 473–482. 18250201 10.1083/jcb.200711136 PMC2234226

[12] Szymanski KM, Binns D, Bartz R, Grishin NV, Li WP, et al (2007) The lipodystrophy protein seipin is found at endoplasmic reticulum lipid droplet junctions and is important for droplet morphology. Proc Natl Acad Sci U S A 104: 20890–20895. 18093937 10.1073/pnas.0704154104 PMC2409237

[13] Windpassinger C, Auer-Grumbach M, Irobi J, Patel H, Petek E, et al (2004) Heterozygous missense mutations in BSCL2 are associated with distal hereditary motor neuropathy and Silver syndrome. Nat Genet 36: 271–276. 14981520 10.1038/ng1313

[14] Ito D, Fujisawa T, Iida H, Suzuki N (2008) Characterization of seipin/BSCL2, a protein associated with spastic paraplegia 17. Neurobiol Dis 31: 266–277. 18585921 10.1016/j.nbd.2008.05.004

[15] Cui X, Wang Y, Tang Y, Liu Y, Zhao L, et al (2011) Seipin ablation in mice results in severe generalized lipodystrophy. Hum Mol Genet 20: 3022–3030. 21551454 10.1093/hmg/ddr205

[16] Yagi T, Ito D, Nihei Y, Ishihara T, Suzuki N (2011) N88S seipin mutant transgenic mice develop features of seipinopathy/BSCL2-related motor neuron disease via endoplasmic reticulum stress. Hum Mol Genet 20: 3831–3840. 21750110 10.1093/hmg/ddr304

[17] Tian Y, Bi J, Shui G, Liu Z, Xiang Y, et al (2011) Tissue-autonomous function of Drosophila seipin in preventing ectopic lipid droplet formation. PLoS Genet 7: e1001364. 21533227 10.1371/journal.pgen.1001364 PMC3077376

[18] Wolinski H, Kolb D, Hermann S, Koning RI, Kohlwein SD (2011) A role for seipin in lipid droplet dynamics and inheritance in yeast. J Cell Sci 124: 3894–3904. 22100922 10.1242/jcs.091454

[19] Cowley MA, Smart JL, Rubinstein M, Cerdan MG, Diano S, et al (2001) Leptin activates anorexigenic POMC neurons through a neural network in the arcuate nucleus. Nature 411: 480–484. 11373681 10.1038/35078085

[20] Ito D, Suzuki N (2007) Molecular pathogenesis of seipin/BSCL2-related motor neuron diseases. Ann Neurol 61: 237–250. 17387721 10.1002/ana.21070

[21] Silver JR (1966) Familial spastic paraplegia with amyotrophy of the hands. Ann Hum Genet 30: 69–75. 5964029 10.1111/j.1469-1809.1966.tb00007.x

[22] Ahima RS (2006) Adipose tissue as an endocrine organ. Obesity (Silver Spring) 14 Suppl 5242S–249S. 17021375 10.1038/oby.2006.317

[23] Sanchez-Lasheras C, Konner AC, Bruning JC (2010) Integrative neurobiology of energy homeostasis-neurocircuits, signals and mediators. Front Neuroendocrinol 31: 4–15. 19729032 10.1016/j.yfrne.2009.08.002

[24] Gautron L, Elmquist JK (2011) Sixteen years and counting: an update on leptin in energy balance. J Clin Invest 121: 2087–2093. 21633176 10.1172/JCI45888 PMC3104762

[25] Bartness TJ, Shrestha YB, Vaughan CH, Schwartz GJ, Song CK (2010) Sensory and sympathetic nervous system control of white adipose tissue lipolysis. Mol Cell Endocrinol 318: 34–43. 19747957 10.1016/j.mce.2009.08.031 PMC2826518

[26] Wang L, Barachina MD, Martinez V, Wei JY, Tache Y (2000) Synergistic interaction between CCK and leptin to regulate food intake. Regul Pept 92: 79–85. 11024569 10.1016/s0167-0115(00)00153-1

[27] Blevins JE, Schwartz MW, Baskin DG (2004) Evidence that paraventricular nucleus oxytocin neurons link hypothalamic leptin action to caudal brain stem nuclei controlling meal size. Am J Physiol Regul Integr Comp Physiol 287: R87–96. 15044184 10.1152/ajpregu.00604.2003

[28] Potes CS, Turek VF, Cole RL, Vu C, Roland BL, et al (2010) Noradrenergic neurons of the area postrema mediate amylin’s hypophagic action. Am J Physiol Regul Integr Comp Physiol 299: R623–631. 20554938 10.1152/ajpregu.00791.2009

[29] Biag J, Huang Y, Gou L, Hintiryan H, Askarinam A, et al (2011) Cyto- and chemoarchitecture of the hypothalamic paraventricular nucleus in the C57BL/6J male mouse: a study of immunostaining and multiple fluorescent tract tracing. J Comp Neurol 520: 6–33. 10.1002/cne.22698 PMC4104804 21674499

[30] Simmons DM, Swanson LW (2009) Comparison of the spatial distribution of seven types of neuroendocrine neurons in the rat paraventricular nucleus: toward a global 3D model. J Comp Neurol 516: 423–441. 19655400 10.1002/cne.22126

[31] Atasoy D, Betley JN, Su HH, Sternson SM (2012) Deconstruction of a neural circuit for hunger. Nature. 10.1038/nature11270 PMC3416931 22801496

[32] Appleyard SM, Marks D, Kobayashi K, Okano H, Low MJ, et al (2007) Visceral afferents directly activate catecholamine neurons in the solitary tract nucleus. J Neurosci 27: 13292–13302. 18045923 10.1523/JNEUROSCI.3502-07.2007 PMC6673415

[33] Cui RJ, Li X, Appleyard SM (2011) Ghrelin inhibits visceral afferent activation of catecholamine neurons in the solitary tract nucleus. J Neurosci 31: 3484–3492. 21368060 10.1523/JNEUROSCI.3187-10.2011 PMC3163901

[34] Petrov T, Krukoff TL, Jhamandas JH (1993) Branching projections of catecholaminergic brainstem neurons to the paraventricular hypothalamic nucleus and the central nucleus of the amygdala in the rat. Brain Res 609: 81–92. 8099526 10.1016/0006-8993(93)90858-k

[35] Maejima Y, Sedbazar U, Suyama S, Kohno D, Onaka T, et al (2009) Nesfatin-1-regulated oxytocinergic signaling in the paraventricular nucleus causes anorexia through a leptin-independent melanocortin pathway. Cell Metab 10: 355–365. 19883614 10.1016/j.cmet.2009.09.002

[36] Garg A (2000) Lipodystrophies. Am J Med 108: 143–152. 11126308 10.1016/s0002-9343(99)00414-3

[37] Payne VA, Grimsey N, Tuthill A, Virtue S, Gray SL, et al (2008) The human lipodystrophy gene BSCL2/seipin may be essential for normal adipocyte differentiation. Diabetes 57: 2055–2060. 18458148 10.2337/db08-0184 PMC2494687

[38] Agostini M, Schoenmakers E, Mitchell C, Szatmari I, Savage D, et al (2006) Non-DNA binding, dominant-negative, human PPARgamma mutations cause lipodystrophic insulin resistance. Cell Metab 4: 303–311. 17011503 10.1016/j.cmet.2006.09.003 PMC1821092

[39] Sarruf DA, Yu F, Nguyen HT, Williams DL, Printz RL, et al (2009) Expression of peroxisome proliferator-activated receptor-gamma in key neuronal subsets regulating glucose metabolism and energy homeostasis. Endocrinology 150: 707–712. 18845632 10.1210/en.2008-0899 PMC2646542

[40] Lu M, Sarruf DA, Talukdar S, Sharma S, Li P, et al (2011) Brain PPAR-gamma promotes obesity and is required for the insulin-sensitizing effect of thiazolidinediones. Nat Med 17: 618–622. 21532596 10.1038/nm.2332 PMC3380629

